# AI-Based Automated Segmentation of the Mandibular Cortex and Mental Foramen for Radiomorphometric Measurement on Panoramic Radiographs: A Feasibility Study

**DOI:** 10.3390/diagnostics16203395

**Published:** 2026-10-19

**Authors:** Sinem Atay Görür, Merve Önder, Özer Çelik, İbrahim Şevki Bayrakdar, Sérgio Lúcio Pereira de Castro Lopes, Andre Luiz Ferreira Costa, Kaan Orhan

**Affiliations:** 1 Dentiron Dental Clinic, 06610 Ankara, Turkey; sinemtygrr@gmail.com; 2 Department of Oral and Maxillofacial Radiology, Faculty of Dentistry, Ankara University, 06500 Ankara, Turkey; merveonder_16@hotmail.com; 3 Department of Mathematics and Computer Science, Faculty of Science, Anadolu University, 26470 Eskişehir, Turkey; ozercelik@anadolu.edu.tr; 4 Department of Oral and Maxillofacial Radiology, Faculty of Dentistry, Eskişehir Osmangazi University, 26040 Eskişehir, Turkey; ibrahimsevkibayrakdar@gmail.com; 5 Department of Diagnosis and Surgery, The Institute of Sciences and Technology, São Paulo State University (UNESP), São José dos Campos 12245-000, SP, Brazil; sergio.lopes@unesp.br; 6 Postgraduate Program in Dentistry, Dentomaxillofacial Radiology and Imaging Laboratory, Cruzeiro do Sul University (UNICSUL), São Paulo 01506-000, SP, Brazil; alfcosta@gmail.com; 7 Department of Oral Diagnostics, Faculty of Dentistry, Semmelweis University, 1088 Budapest, Hungary; 8 Medical Design Application and Research Center (MEDITAM), Ankara University, 06520 Ankara, Turkey

**Keywords:** artificial intelligence, deep learning, YOLOv5, panoramic radiograph, mandibular cortical width, panoramic mandibular index, osteoporosis, opportunistic screening

## Abstract

**Background/Objectives**: This study evaluated the feasibility of a YOLOv5-based model for automated segmentation of these structures and automated measurement of mandibular cortical width (MCW) and panoramic mandibular index PMI. **Methods**: Five hundred panoramic radiographs were retrospectively collected. The mandibular cortex and the right and left mental foramina were annotated with polygon labels as three separate classes and verified by an experienced radiologist; intra- and inter-observer reliability was evaluated on a random 10% subset. A YOLOv5s-seg model was trained for 500 epochs and evaluated on an independent, patient-level test set. Model performance was quantified with precision, recall, F1 score, Dice coefficient, mean intersection over union (mIoU), and mean average precision (mAP@0.5 and mAP@0.5:0.95). **Results**: The model achieved a precision of 0.94, a recall of 0.82, and an F1 score of 0.88 (mAP@0.5 = 0.86; mAP@0.5:0.95 = 0.52). The Dice coefficient was 0.93 for the mandibular cortex and 0.80 and 0.75 for the right and left mental foramina, respectively. In the side-level paired agreement analysis (62 measurable sides from 44 test-set patients), the ICC was 0.89 (95% CI: 0.84–0.93) for MCW, with a mean bias of +0.08 mm and 0.87 for PMI, with a mean bias of +0.01. **Conclusions**: YOLOv5 showed high agreement with expert observers for mandibular cortex segmentation and moderate agreement for the mental foramen, supporting the technical feasibility of automated MCW and PMI measurement. Because missed mental foramen detections reduce the number of measurable sites, automated measurement should be combined with a verification step.

## 1. Introduction

Osteoporosis is a common metabolic bone disease and a major public health problem, particularly in older adults. Because the disease is often clinically silent until a fragility fracture occurs, vertebral, hip, and wrist fractures are frequently its first manifestation; hip fracture is associated with substantial mortality and loss of independence. Early identification of individuals at risk is therefore a clinical priority [1,2].

Dual-energy X-ray absorptiometry (DXA) is the reference standard for measuring bone mineral density (BMD). However, underdiagnosis remains common [2]. Dental radiographs are obtained frequently, are inexpensive, and are already digitized in most practices, which makes them attractive for opportunistic screening [3]. Panoramic radiographs visualize both jaws and the inferior mandibular cortex, where osteoporosis-related changes can be observed [4]. As a two-dimensional modality, panoramic imaging has inherent limitations, including magnification, distortion, and superimposition; accordingly, its appropriate role is to support the identification of at-risk individuals who should be referred for DXA, rather than to replace a definitive diagnostic method [3,5].

Among the radiomorphometric indices proposed on panoramic radiographs, the mandibular cortical width (MCW) and the panoramic mandibular index (PMI) are the most widely studied, and both have shown correlations with DXA-derived T-scores [6,7]. Accurate measurement of these indices depends on precise localization of the mental foramen and the inferior border of the mandibular cortex. In most published systems, these structures are marked manually, which limits reproducibility and hinders integration into the clinical workflow [8].

Deep learning-based segmentation offers a potential solution. Convolutional neural networks have become the dominant approach in medical image analysis [9,10], and the You Only Look Once (YOLO) family of models is widely used for object detection, and in its segmentation variants, for polygon-based delineation [11]. Two-dimensional convolutional neural networks have repeatedly been shown to perform well for anatomical labeling in dental imaging [12], and the need for automatic localization of the mental foramen before MCW measurement has been explicitly emphasized [13]. More recently (2024–2025), deep learning has been applied on panoramic radiographs for tooth detection and segmentation with YOLO-based models [14], for osteoporosis detection focused on the mental foramen region [15], and in systematic reviews and meta-analyses confirming the diagnostic potential of these approaches [16].

The aim of this study was therefore to evaluate the feasibility of a YOLOv5-based model for the automated segmentation of the mandibular cortex and the mental foramen on panoramic radiographs, and for the automated measurement of MCW and PMI, using expert observer annotations as the reference standard. This study was designed and is reported as a feasibility study of anatomical labeling and measurement; it does not evaluate the diagnostic accuracy of the model for osteoporosis, for which a bone mineral density reference standard would be required.

## 2. Materials and Methods

### 2.1. Study Design and Image Dataset

This retrospective study was conducted in accordance with the Declaration of Helsinki and was approved by the Ethics Committee of Ankara University Faculty of Dentistry Clinical Research (approval number 36290600; decision number 52/2021; approval date: 14 April 2021). Due to the retrospective design, informed consent was waived by the ethics committee.

Panoramic radiographs previously obtained for various dental indications from patients aged 18–80 years at the Department of Oral, Dental, and Maxillofacial Radiology, Ankara University Faculty of Dentistry, were retrospectively screened. Five hundred consecutive eligible images were included. The final dataset consisted of 500 panoramic radiographs: 200 from patients with a documented diagnosis of osteoporosis in their medical records and 300 from patients without a known osteoporosis diagnosis. No DXA measurements were available for this dataset; therefore, the osteoporosis-related group assignment was based solely on existing medical records and was used only for descriptive purposes, not for any diagnostic analysis.

Consecutive sampling was applied: all eligible examinations recorded during the study period that met the criteria were included in chronological order until the target sample size was reached. The proportion of patients with documented osteoporosis (40%) reflects the referral profile of a university-based dentomaxillofacial radiology service, which serves a comparatively old patient population with a high proportion of postmenopausal women referred for implant planning and bone assessment; because this grouping was used only descriptively and no diagnostic analysis was performed, it does not introduce spectrum bias into the performance estimates reported here.

**Figure 1:**
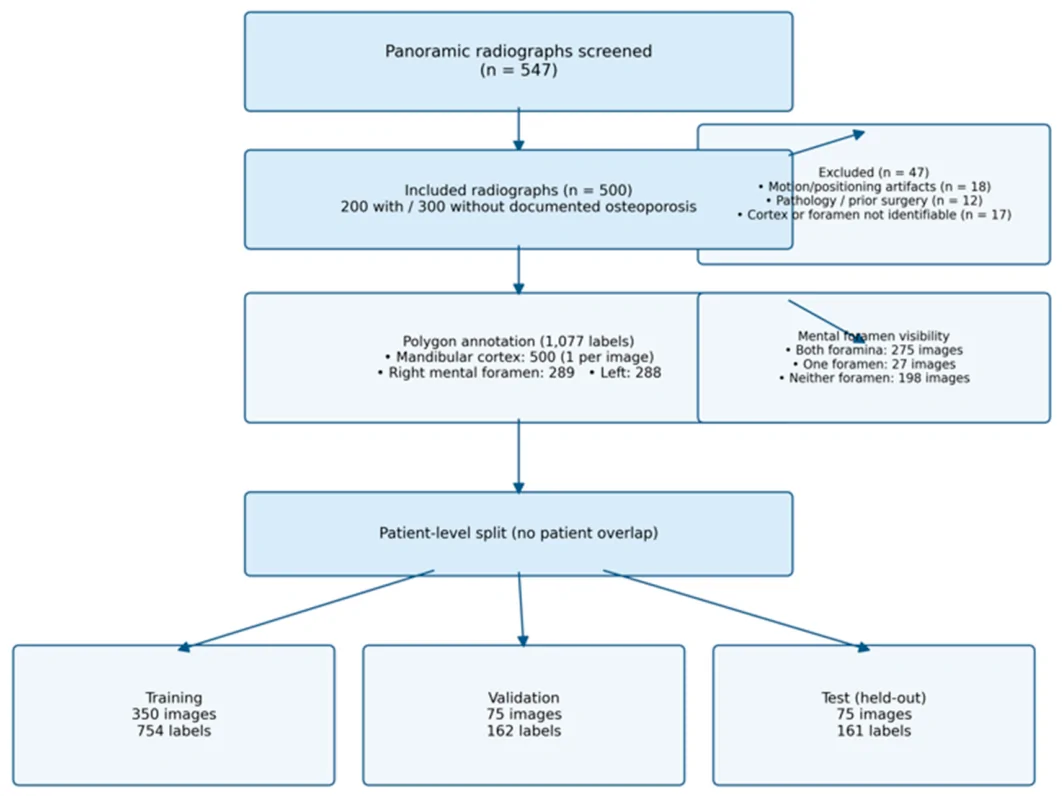
Flow diagram of image selection, annotation, and dataset partitioning.

Inclusion criteria were: (i) high-quality panoramic radiographs with complete visualization of the inferior border of the mandibular cortex bilaterally; and (ii) patients aged 18–80 years. Mental foramina were annotated whenever they could be reliably identified; however, identifiability of both mental foramina was not an inclusion requirement because the mental foramen is not radiographically distinguishable in a considerable proportion of panoramic radiographs. Exclusion criteria were: images with motion or positioning artifacts affecting the region of interest, pathological lesions or previous surgery/fracture in the mandibular body region, and images in which the inferior mandibular cortex could not be reliably identified. Of the 547 images screened, 47 were excluded (18 motion/positioning artifacts, 12 pathological lesions or prior surgery, 17 unidentifiable mental foramen or cortex), leaving 500 images for analysis. Among the 500 included images, both mental foramina could be annotated in 275 images, only one mental foramen in 27 images (right only in 14, left only in 13), and neither mental foramen in 198 images. The complete selection and annotation flow is summarized in Figure 1.

### 2.2. Reference Annotations and Index Definitions

All anatomical structures were labeled by a single trained observer using polygon-based (freehand) annotation in Colabeler software (Eskişehir, Turkey). Three annotation classes were defined: the mandibular cortex (a single polygon per image covering the inferior cortex bilaterally), the right mental foramen, and the left mental foramen. A total of 1077 valid labels were obtained: 500 mandibular cortices, 289 right mental foramina, and 288 left mental foramina. All labels were verified by an oral and maxillofacial radiologist with 20 years of experience, and discrepancies were resolved by consensus. Images or structures that could not be labeled reliably were excluded from the corresponding analyses. All observer annotations and measurements were performed before model development and were blinded to the model outputs.

To quantify observer-related measurement error, a random subset of 50 images (10% of the dataset) was re-annotated and re-measured by the primary observer after a 4-week washout period (intra-observer reliability) and was independently annotated and measured by the verifying radiologist (inter-observer reliability). Intra-observer ICCs were 0.94 (95% CI: 0.90–0.97) for MCW and 0.92 (95% CI: 0.86–0.95) for PMI; inter-observer ICCs were 0.91 (95% CI: 0.85–0.95) for MCW and 0.89 (95% CI: 0.81–0.93) for PMI, indicating excellent reproducibility of the reference measurements.

**Figure 2:**
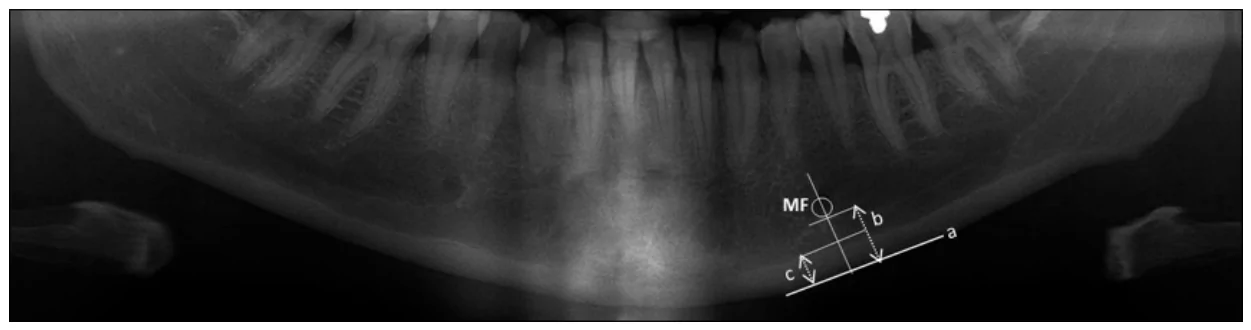
Schematic definition of the mandibular cortical width (c) and the distance used for the panoramic mandibular index (b). A tangent line is drawn along the inferior border of the mandible at the level of the mental foramen (line a), and a perpendicular line is drawn through the mental foramen. MCW is measured between the superior and inferior borders of the cortex at the intersection point (line c).

**Figure 3:**
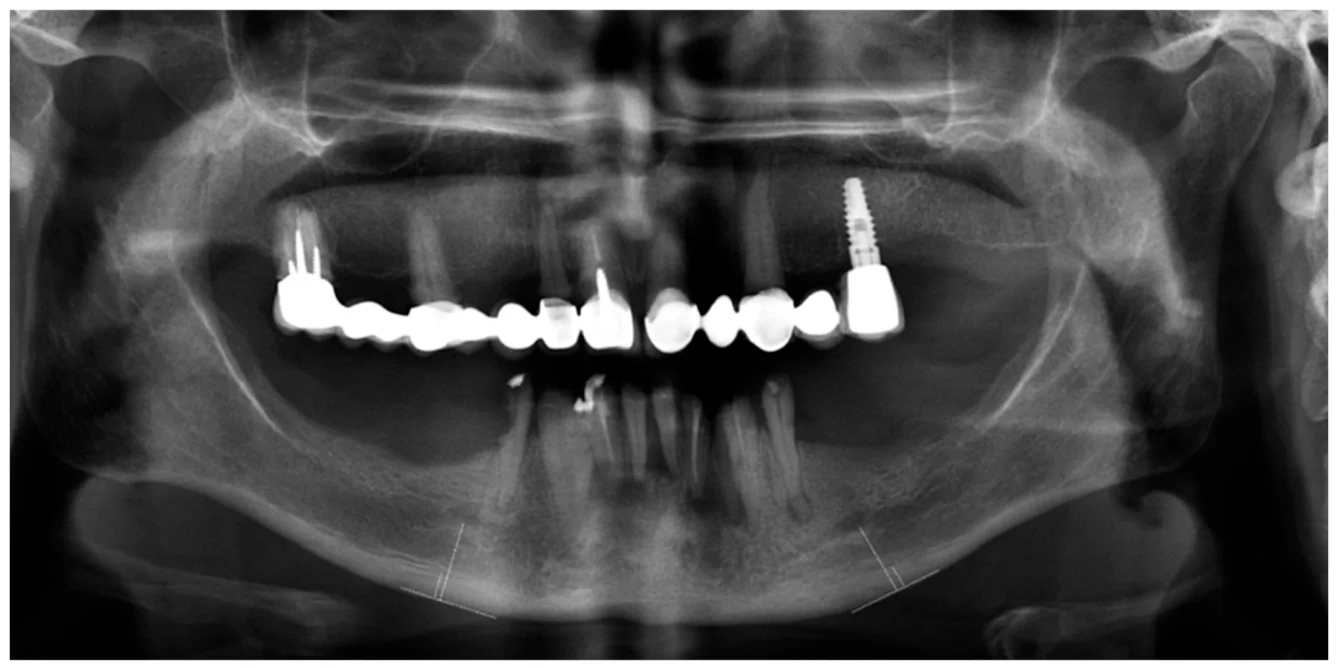
Measurement of MCW and PMI with ImageJ software. Scale calibration was applied using the known pixel spacing for each imaging device.

Mandibular cortical width (MCW) was defined as the thickness of the mandibular cortex just distal to the mental foramen. A tangent line was drawn along the inferior border of the mandible at the level of the mental foramen, and a perpendicular line was drawn through the mental foramen (Figure 2, line b). MCW was measured between the superior and inferior borders of the cortex at the intersection point (Figure 2, line c). The panoramic mandibular index (PMI) was calculated as the ratio c/b. Both indices were measured separately on the right and left sides and averaged. Reference measurements were performed with ImageJ software (version 1.53k, National Institutes of Health, Bethesda, MD, USA) and verified by an experienced radiologist (Figure 3). A pixel-to-millimetre calibration factor was applied for each image using the manufacturer’s stated pixel spacing; this was possible for 462 out of the 500 images (92.4%); for the remaining 38 images (7.6%) acquired on devices without available pixel-spacing metadata, a fixed calibration was derived from the known diameter of the positioning bite rod visible in the image.

### 2.3. Deep Learning Model and Training

Polygon annotations were exported from Colabeler as JSON files containing the absolute pixel coordinates of the polygon vertices and were converted to the YOLO segmentation format with a custom Python script, which normalizes the vertex coordinates by the image width and height and pairs each polygon with its class index (0 = mandibular cortex, 1 = right mental foramen, 2 = left mental foramen). The conversion script is available from the corresponding author upon reasonable request. A YOLOv5s-seg model (small variant, Ultralytics repository, version 7.0) [11] was trained for 500 epochs. YOLOv5 was selected for the following reasons. First, at the time of model development, version 7.0 was a mature, stable, and extensively documented release with an integrated instance-segmentation head, whereas newer iterations (YOLOv8 through YOLO12) had limited validation in dentomaxillofacial segmentation tasks. Second, the small variant offers a favorable balance between accuracy and computational cost, enabling inference on standard clinical workstations, which is relevant to the intended chair-side screening scenario. Third, YOLOv5-based pipelines have repeatedly demonstrated strong performance in panoramic dental segmentation tasks [11,14], allowing for a direct comparison with the existing literature. A systematic benchmarking against newer architectures is planned as future work. The input images were resized to 640 × 640 pixels with letterbox padding so that the original aspect ratio was preserved and no anisotropic stretching was introduced; predicted polygon coordinates were mapped back to the original image dimensions through the inverse letterbox transformation before any measurement, and all measurements were therefore performed in the native image space. Images were normalized to the [0, 1] range; grayscale images were converted to three-channel RGB by channel duplication. The following data augmentations were applied during training: mosaic (probability 1.0), random horizontal flip (probability 0.5), HSV hue shift (±1.5%), HSV saturation gain (±70%), HSV value gain (±40%), random translation (±10%), and random scaling (±50%). No rotation, shear, perspective distortion, or vertical flip was used. Training hyperparameters were: Stochastic Gradient Descent optimizer, initial learning rate 0.01, final learning rate 0.0001 (cosine annealing), momentum 0.937, weight decay 0.0005, and batch size 16. The model was trained on a workstation equipped with an NVIDIA GeForce RTX 3090 GPU (24 GB VRAM) using Python 3.9.12, PyTorch 1.12.1, CUDA 11.6, and Ubuntu 20.04. The best checkpoint was selected based on the lowest validation segmentation loss. Reporting of the deep learning pipeline follows available checklists for artificial intelligence research in dentistry [17].

For automated measurement, the predicted polygon for the mandibular cortex was converted to a binary mask. To reproduce the local tangent used in the conventional manual definition, a least-squares line was fitted to the inferior border pixels of the mask within a local window extending 10 mm on either side of the mental foramen, rather than to the entire inferior border. The mental foramen centroid was computed from its predicted polygon. A perpendicular line from the centroid to the fitted tangent was constructed, and the cortical thickness (MCW) was measured along this perpendicular by counting mask pixels between the superior and inferior cortical borders and converting to millimetres using the image-specific calibration factor. PMI was computed as the ratio of the calibrated MCW to the calibrated perpendicular distance from the foramen centroid to the tangent line. Model-derived measurements were thus based on the predicted (not ground-truth) mental foramen centroids; sides on which the model failed to detect the mental foramen did not yield an automated measurement and were excluded from the agreement analysis (complete-case approach, Section 2.5).

### 2.4. Dataset Partitioning

The dataset was divided at the patient level into training, validation, and test subsets, with no patient appearing in more than one subset. The split ratios were 70% training (350 images, 754 labels), 15% validation (75 images, 162 labels), and 15% test (75 images, 161 labels). The distribution of images and annotations per anatomical structure across the three subsets is summarized in Table 1. Model development, including epoch selection and early-stopping monitoring, used only the training and validation subsets. All reported performance metrics were computed once on the held-out test subset.

### 2.5. Performance Evaluation and Statistical Analysis

Because the task performed by the model was anatomical structure detection rather than patient-level classification, performance was evaluated at the object level using precision (TP/(TP + FP)), recall (TP/(TP + FN)), and the F1 score (2TP/(2TP + FP + FN)) at an intersection-over-union (IoU) threshold of 0.5. Segmentation quality was additionally quantified with the Dice coefficient and mean IoU. Mean average precision at an IoU threshold of 0.5 (mAP@0.5) and averaged over IoU thresholds from 0.5 to 0.95 in steps of 0.05 (mAP@0.5:0.95) are also reported. Precision–recall curves for each class and the training/validation learning curves are provided as Supplementary Figures S1 and S2, respectively. Image-level accuracy and ROC/AUC analysis were not performed, because no patient-level osteoporosis classification task was carried out; in object detection, true negatives (background regions correctly not flagged) are not enumerated, and accuracy/AUC values computed from detection counts are not meaningful.

Agreement between observer- and model-derived MCW and PMI measurements was assessed using the intraclass correlation coefficient (ICC, two-way random effects, absolute agreement, single measure) and Bland–Altman analysis (mean bias and 95% limits of agreement). Agreement analyses were performed at the side level and included all sides for which both an observer annotation and a corresponding model detection of the mental foramen were available (paired complete-case analysis). Sides on which the model failed to detect the mental foramen (9 right and 15 left) did not yield an automated measurement and were therefore excluded from the agreement analysis; the exact sample size of each analysis is reported together with the results. In a secondary analysis, right and left values were averaged for the 18 patients in whom both sides were measurable to confirm that the averaging approach did not alter the findings. The Shapiro–Wilk test was used to check normality. Statistical analyses were performed using SPSS version 26.0 (IBM Corp., Armonk, NY, USA) and Python 3.9 with the SciPy and Pingouin libraries. A *p*-value < 0.05 was considered statistically significant.

## 3. Results

### 3.1. Dataset and Demographic Characteristics

The demographic and clinical characteristics of the 500 included patients are summarized in Table 2. The mean age was 52.3 ± 14.7 years (range 18–80 years). The sample comprised 320 females (64.0%) and 180 males (36.0%). Among the female patients, 210 (65.6%) were postmenopausal. Two hundred patients (40.0%) had a documented osteoporosis diagnosis in their medical records, whereas 300 (60.0%) did not. The group with documented osteoporosis was significantly older than the group without (61.4 ± 9.2 years versus 46.2 ± 12.8 years; *p* < 0.001).

### 3.2. Segmentation Performance

Segmentation overlap with the observer annotations was high for the mandibular cortex (Dice = 0.93; mIoU = 0.87) and moderate for the mental foramen (Dice = 0.80 and 0.75; mIoU = 0.67 and 0.60 for the right and left sides, respectively). On the independent test set (161 annotated structures from 75 patients), the model achieved an overall precision of 0.94, a recall of 0.82, and an F1 score of 0.88 (mAP@0.5 = 0.86; mAP@0.5:0.95 = 0.52) (Table 3). These values were computed directly from the object-level counts (true positives = 132, false positives = 8, false negatives = 29). The precision–recall curves for the three classes (Supplementary Figure S1) were consistent with these point estimates, and the training/validation learning curves (Supplementary Figure S2) showed stable convergence.

Per-structure performance is detailed in Table 3. The mandibular cortex was detected and segmented with higher precision, recall, and Dice coefficient than the mental foramen. Between the right and left mental foramina, performance was comparable, with slightly lower recall on the left side.

**Figure 4:**
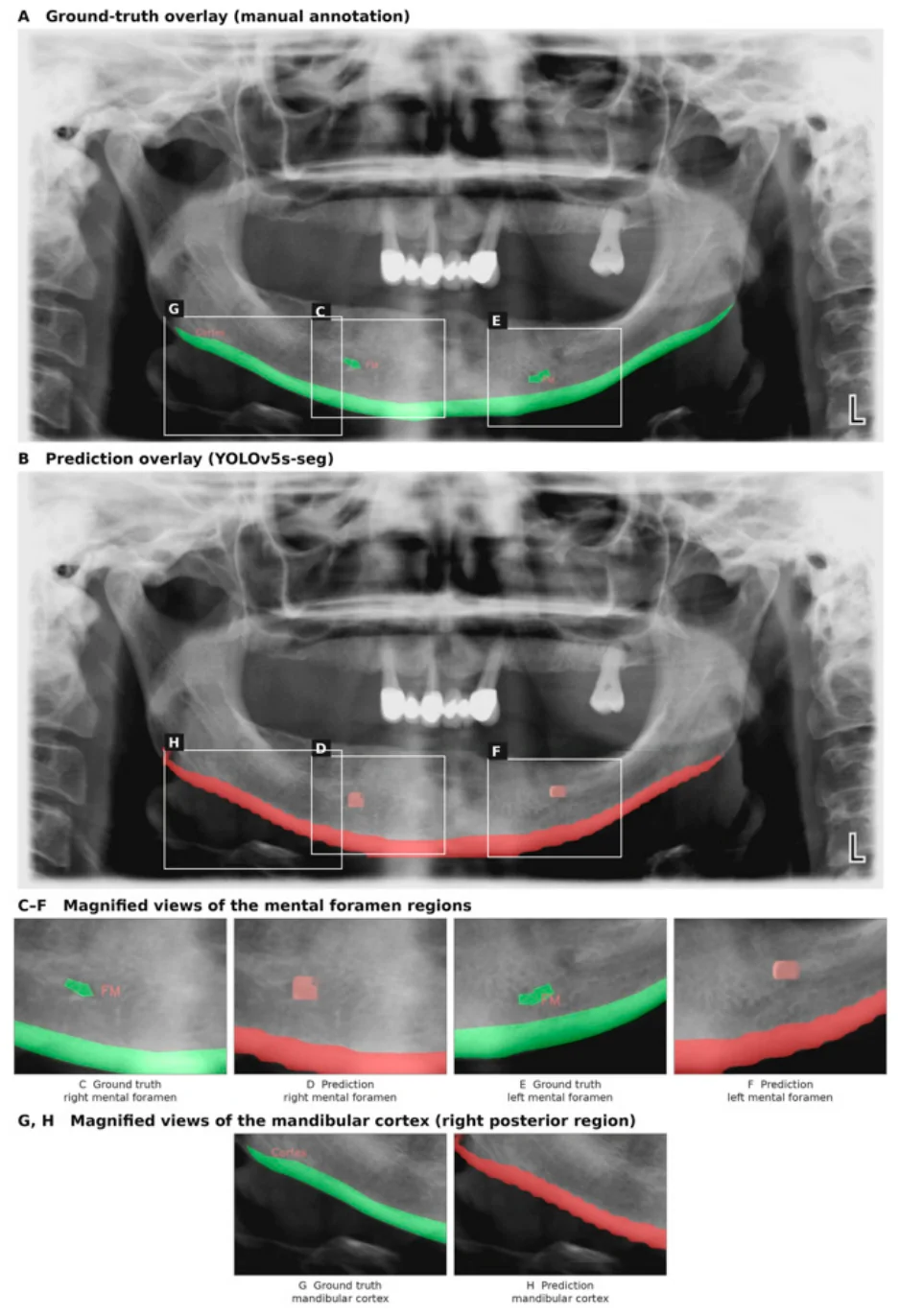
Qualitative comparison of ground-truth and predicted instance segmentation on a representative test-set panoramic radiograph. (**A**) Original radiograph overlaid with the manual ground-truth annotations (green): the mandibular cortex and both mental foramina. (**B**) The same radiograph overlaid with the YOLOv5s-seg prediction (red): the predicted mandibular cortex band and both mental foramen regions. (**C**–**F**) Magnified views of the right (**C**,**D**) and left (**E**,**F**) mental foramen regions ((**C**,**E**): ground truth; (**D**,**F**): prediction). (**G**,**H**) Magnified views of the mandibular cortex in the right posterior region ((**G**) ground truth; (**H**) prediction). All overlays are pixel-level segmentation masks.

Qualitative inspection of the test-set predictions (Figure 4) showed that missed or under-segmented mental foramina were predominantly associated with superimposition of the mandibular canal or root apices, low local contrast between the foramen and the surrounding trabecular bone, and the small size of the structure relative to the full image.

The segmentation loss decreased steadily over the first 300 epochs and plateaued thereafter, with no substantial divergence between training and validation loss, suggesting that the model did not overfit severely to the training set (Supplementary Figure S2).

### 3.3. Agreement of Model-Derived and Observer-Derived Measurements

Automated MCW and PMI measurements require a detected mental foramen; therefore, the agreement analysis included the 62 sides (32 right and 30 left) from 44 test-set patients for which both an observer annotation and a corresponding model detection were available; 18 of these patients had bilateral paired measurements. The mean mandibular cortical width measured by the observer was 3.82 ± 0.74 mm on the right side and 3.79 ± 0.71 mm on the left side; the corresponding model-derived values were 3.91 ± 0.78 mm and 3.88 ± 0.75 mm (Table 4). The ICC for MCW between the observer and model was 0.89 (95% CI: 0.84–0.93). Bland–Altman analysis showed a mean bias of +0.08 mm with 95% limits of agreement from-1.12 mm to+1.28 mm. The secondary analysis based on right–left averaged values in the 18 patients with bilateral measurements yielded a nearly identical ICC (0.90; 95% CI: 0.76–0.96).

For the panoramic mandibular index, the observer-derived mean was 0.32 ± 0.05 on the right side and 0.32 ± 0.05 on the left side; the model-derived means were 0.33 ± 0.05 and 0.33 ± 0.05 (Table 4). The ICC for PMI was 0.87 (95% CI: 0.81–0.91). Bland–Altman analysis showed a mean bias of 0.01 with 95% limits of agreement from -0.08− to +0.10.

## 4. Discussion

In this study, a YOLOv5 segmentation model was evaluated for the automated delineation of the mandibular cortex and the mental foramen on panoramic radiographs and for the automated measurement of MCW and PMI. The principal finding was that the model achieved high overlap with expert observers for the mandibular cortex (Dice = 0.93) and moderate overlap for the mental foramen (Dice = 0.75–0.80), with an overall object-level precision of 0.94, recall of 0.82, and F1 score of 0.88. Measurement agreement was also high, with ICC values of 0.89 for MCW and 0.87 for PMI. These findings support the technical feasibility of automated radiomorphometric measurement on panoramic radiographs.

The lower agreement observed for the mental foramen is anatomically plausible. The mental foramen shows interindividual variability in position and shape, may be partially superimposed on the mandibular canal or root apices on two-dimensional images, and has less consistent borders than the inferior mandibular cortex [18]. Our Dice values for the mental foramen (0.75–0.80) are comparable to those reported in a recent dedicated segmentation study, in which a UNet model trained on 1000 panoramic radiographs reached a Dice coefficient of 0.79 for the mental foramen [18], suggesting that the moderate performance observed here reflects the intrinsic difficulty of the structure rather than a deficiency of the specific model. This interpretation is also consistent with previous reports emphasizing that automatic localization of the mental foramen is the key unmet need before MCW can be measured automatically [13].

Our findings are in line with previous work on radiomorphometric indices and computer-aided analysis. Taguchi et al. and Devlin and Horner reported that panoramic radiographs may assist in identifying patients with low BMD [3,5], and Triantafyllopoulos et al. found significant correlations between MCW and DXA T-scores, suggesting referral for DXA when cortical thickness is below 3 mm [19]. Abdinian et al. reported significant correlations between PMI and spinal and femoral T-scores [20]. Arifin et al. measured MCW with computer assistance but required radiologists to mark the mental foramen manually, and explicitly called for a system that labels the mental foramen automatically [13]. Kavitha et al. reported high sensitivity and specificity for a support vector machine approach in postmenopausal women [21]. More recent studies have shifted from hand-crafted indices to end-to-end deep learning: Sukegawa et al. identified osteoporosis using an ensemble model combining panoramic radiographs with clinical covariates [22]; Gaudin et al. focused a deep learning classifier on the mental foramen region and demonstrated that apparently high accuracies can arise from age-related confounding [15]; and two recent meta-analyses reported pooled sensitivities of approximately 80% and specificities of 87–92% for deep learning-based osteoporosis prediction on plain radiographs and panoramic images [16,23]. Kiswanjaya et al. confirmed that MCW decreases significantly with age and is lower in patients with cortical erosion, underlining the importance of age as a confounder in this research field [24]. Compared with these studies, the present work addresses the automation step itself—namely segmentation and measurement—rather than disease classification; however, unlike classification studies, it does not—and does not claim to—establish diagnostic accuracy against a BMD reference standard.

The clinical acceptability of automated measurements must be judged against the thresholds used in practice. For MCW, the 95% limits of agreement (−1.12 to +1.28 mm) are not negligible relative to the commonly proposed 3 mm referral threshold [19]: for patients whose true MCW lies within approximately 1.2 mm of the threshold, observer–model disagreement of this magnitude could shift the screening classification in either direction. The system should therefore be regarded as a triage aid whose output prompts expert review rather than as a stand-alone decision tool, and borderline measurements should be verified manually. For PMI, the limits of agreement of −0.08 to +0.10 around a mean value of approximately 0.32 correspond to a relative difference of roughly 25–30%; however, this magnitude is of the same order as the intra- and inter-observer variability of manual PMI measurement, and PMI is conventionally interpreted as an adjunctive ratio rather than a stand-alone criterion [5,19].

The failure analysis provides directions for future improvement. Missed or under-segmented mental foramina were mainly associated with superimposition of the mandibular canal or root apices, low local contrast, and the small relative size of the foramen. Possible countermeasures include training at higher input resolution or on tiled image patches, attention-based or two-stage detection architectures, enlarging the mental foramen annotation set with hard-negative mining, and multi-center training data to cover device- and population-related variability [15,18,25]. Because sides without a model detection did not contribute to the agreement analysis, the reported ICC and Bland–Altman values describe measurement quality conditional on successful detection; in a deployed workflow, undetected foramina would be flagged for manual measurement, so that missed detections reduce the coverage of the automated pipeline rather than silently producing erroneous values.

Clinically, the realistic role of such a system is to support opportunistic screening: panoramic radiographs are routinely obtained in dental practice, and an automated, reproducible MCW/PMI measurement could help dentists identify patients who might benefit from referral for DXA [3,26]; longitudinal data linking skeletal bone loss to oral bone changes further support this concept [27]. Automated anatomical labeling must not be conflated with osteoporosis diagnosis; accurate detection of the mental foramen and mandibular cortex is a prerequisite for measurement, not evidence of diagnostic performance.

### Limitations

This study has several limitations. First, it was a retrospective, single-center study, and the findings may not generalize to other imaging devices, acquisition protocols, or populations. Second, although intra- and inter-observer reliability was excellent, it was evaluated on a 10% subset rather than the full dataset. Third, diagnostic accuracy for osteoporosis was not evaluated because DXA measurements were not available; accordingly, no diagnostic or clinical-applicability claims can be made, and the osteoporosis-related grouping was based on existing medical records only. Fourth, although a patient-level train/test split was used to mitigate data leakage and overfitting, the size of the independent test set was limited. Fifth, agreement for the mental foramen was only moderate, and missed detections (recall of 0.78 and 0.67 for the right and left foramina) reduced the number of sides available for automated measurement, which may introduce a selection effect favoring clearly visible foramina (spectrum bias). Sixth, a single pixel-to-millimetre calibration factor was applied per image, whereas panoramic magnification varies across the image and with patient positioning; and the fitted tangent line is only a local linear approximation of the curved mandibular base. Both factors may contribute to measurement error. Seventh, the groups differed substantially in age, and recent work confirms that age strongly influences MCW [24]; therefore, any model behavior related to the documented osteoporosis group may reflect age-related rather than disease-related features. External, multicenter validation and a study linking model-derived measurements to DXA-derived BMD/T-scores are required before any clinical use can be considered.

## 5. Conclusions

The YOLOv5 model demonstrated high agreement with the expert observers for mandibular cortex segmentation and moderate agreement for the mental foramen on panoramic radiographs, supporting the technical feasibility of automated MCW and PMI measurement. Because automated measurement is conditional on successful mental foramen detection, a verification step for missed or borderline detections is advisable. These results should be interpreted strictly as a feasibility study of anatomical labeling. The model’s ability to contribute to osteoporosis screening can only be established through independent external validation and a formal evaluation against DXA-derived bone mineral density as the reference standard.

## Figures and Tables

**Table 1:** Distribution of patients, radiographs, and annotations for each anatomical structure across the training, validation, and test subsets (patient-level split, no overlap).

Subset	Patients/Images (*n*)	Mandibular Cortex (*n*)	Right Mental Foramen (*n*)	Left Mental Foramen (*n*)	Total Labels (*n*)
Training	350	350	204	200	754
Validation	75	75	44	43	162
Test	75	75	41	45	161
Total	500	500	289	288	1077

**Table 2:** Demographic and clinical characteristics of the study sample (*n* = 500).

Characteristic	Total (*n* = 500)	Documented Osteoporosis * (*n* = 200)	No Documented Osteoporosis * (*n* = 300)
Age (years), mean ± SD	52.3 ± 14.7	61.4 ± 9.2	46.2 ± 12.8
Age range (years)	18–80	28–80	18–79
Sex, *n* (%)			
Female	320 (64.0)	168 (84.0)	152 (50.7)
Male	180 (36.0)	32 (16.0)	148 (49.3)
Postmenopausal women, *n* (% of females)	210 (65.6)	145 (86.3)	65 (42.8)

* Group assignment based on existing medical records; no DXA data were available. SD, standard deviation.

**Table 3:** Object-level detection and segmentation performance of the YOLOv5 model on the independent test set (*n* = 75 images, 161 ground-truth structures). mAP, mean average precision (red columns added in the revision).

Structure	Ground Truth (*n*)	TP	FP	FN	Precision	Recall	F1	Dice	mIoU	mAP@0.5	mAP@0.5:0.95
Mandibular cortex	75	70	2	5	0.97	0.93	0.95	0.93	0.87	0.95	0.68
Right mental foramen	41	32	3	9	0.91	0.78	0.84	0.80	0.67	0.84	0.47
Left mental foramen	45	30	3	15	0.91	0.67	0.77	0.75	0.60	0.79	0.40
Overall (macro)	161	132	8	29	0.94	0.82	0.88	—	—	0.86	0.52

TP, true positive; FP, false positive; FN, false negative; mIoU, mean intersection over union.

**Table 4:** Agreement between the observer- and model-derived MCW and PMI measurements (side-level paired analysis; 62 sides from 44 patients).

Index	Side	Observer, Mean ± SD	Model, Mean ± SD	ICC (95% CI) *	Bland–Altman Bias (95% LoA) *
MCW (mm)	Right (*n* = 32)	3.82 ± 0.74	3.91 ± 0.78	0.89 (0.84–0.93)	+0.08 (−1.12, +1.28)
	Left (*n* = 30)	3.79 ± 0.71	3.88 ± 0.75		
PMI	Right (*n* = 32)	0.32 ± 0.05	0.33 ± 0.05	0.87 (0.81–0.91)	+0.01 (−0.08, +0.10)
	Left (*n* = 30)	0.32 ± 0.05	0.33 ± 0.05		

* ICC calculated across the 62 paired side-level measurements. MCW, mandibular cortical width; PMI, panoramic mandibular index; ICC, intraclass correlation coefficient; CI, confidence interval; LoA, limits of agreement.

## Data Availability

The data supporting this study are available from the corresponding author upon reasonable request.

## Supplementary Materials

The following supporting information can be downloaded at: https://www.mdpi.com/article/10.3390/diagnostics16203395/s1, Figure S1: Precision–recall curves of the YOLOv5s-seg model for the three classes (mandibular cortex, right mental foramen, left mental foramen) on the independent test set at an IoU threshold of 0.5. AP, average precision. Figure S2. Training and validation learning curves over 500 epochs: box, segmentation, and classification losses for the training and validation subsets, together with object-level precision, recall, mAP@0.5, and mAP@0.5:0.95. The segmentation loss decreased steadily over the first ~300 epochs and plateaued thereafter, with no substantial divergence between training and validation loss.

## Abbreviations

The following abbreviations are used in this manuscript:

AUC	Area under the curve
BMD	Bone mineral density
CI	Confidence interval
DXA	Dual-energy X-ray absorptiometry
FN	False negative
FP	False positive
GPU	Graphics processing unit
HSV	Hue, saturation, value
ICC	Intraclass correlation coefficient
IoU	Intersection over union
JSON	JavaScript Object Notation
LoA	Limits of agreement
mAP	Mean average precision
MCW	Mandibular cortical width
mIoU	Mean intersection over union
PMI	Panoramic mandibular index
RGB	Red, green, blue
ROC	Receiver operating characteristic
SD	Standard deviation
TP	True positive
VRAM	Video random-access memory
YOLO	You Only Look Once

## References

[1] Melton L.J., Cooper C., Marcus R., Feldman D., Kelsey J. (2001). Magnitude and impact of osteoporosis and fractures. Osteoporosis.

[2] Chrischilles E.A., Butler C.D., Davis C.S., Wallace R.B. (1991). A model of lifetime osteoporosis impact. Arch. Intern. Med..

[3] Taguchi A., Tanaka R., Kakimoto N., Morimoto Y., Arai Y., Hayashi T., Kurabayashi T., Katsumata A., Asaumi J., Japanese Society for Oral and Maxillofacial Radiology (2021). Clinical guidelines for the application of panoramic radiographs in screening for osteoporosis. Oral Radiol..

[4] Horner K., Devlin H., Alsop C.W., Hodgkinson I.M., Adams J.E. (1996). Mandibular bone mineral density as a predictor of skeletal osteoporosis. Br. J. Radiol..

[5] Devlin H., Horner K. (2002). Mandibular radiomorphometric indices in the diagnosis of reduced skeletal bone mineral density. Osteoporos. Int..

[6] Kribbs P.J., Chesnut C.H., Ott S.M., Kilcoyne R.F. (1990). Relationships between mandibular and skeletal bone in a population of normal women. J. Prosthet. Dent..

[7] Mohajery M., Brooks S.L. (1992). Oral radiographs in the detection of early signs of osteoporosis. Oral Surg. Oral Med. Oral Pathol..

[8] Nakamoto T., Taguchi A., Ohtsuka M., Suei Y., Fujita M., Tsuda M., Sanada M., Kudo Y., Asano A., Tanimoto K. (2008). A computer-aided diagnosis system to screen for osteoporosis using dental panoramic radiographs. Dentomaxillofac. Radiol..

[9] LeCun Y., Bengio Y., Hinton G. (2015). Deep learning. Nature.

[10] Chartrand G., Cheng P.M., Vorontsov E., Drozdzal M., Turcotte S., Pal C.J., Kadoury S., Tang A. (2017). Deep learning: A primer for radiologists. Radiographics.

[11] Jocher G., Chaurasia A., Qiu J. (2022). Ultralytics YOLOv5, Version 7.0.

[12] Lee J.H., Kim D.H., Jeong S.N., Choi S.H. (2018). Detection and diagnosis of dental caries using a deep learning-based convolutional neural network algorithm. J. Dent..

[13] Arifin A.Z., Asano A., Taguchi A., Nakamoto T., Ohtsuka M., Tanimoto K. (2005). Computer-aided system for measuring the mandibular cortical width on panoramic radiographs in osteoporosis diagnosis. Proceedings of SPIE, Medical Imaging 2005.

[14] Beser B., Reis T., Berber M.N., Topaloglu E., Gungor E., Kılıc M.C., Duman S., Çelik Ö., Kuran A., Bayrakdar I.S. (2024). YOLO-V5 based deep learning approach for tooth detection and segmentation on pediatric panoramic radiographs in mixed dentition. BMC Med. Imaging.

[15] Gaudin R., Otto W., Ghanad I., Kewenig S., Rendenbach C., Alevizakos V., Grün P., Kofler F., Heiland M., von See C. (2024). Enhanced osteoporosis detection using artificial intelligence: A deep learning approach to panoramic radiographs with an emphasis on the mental foramen. Med. Sci..

[16] Tarighatnia A., Amanzadeh M., Hamedan M., Mohammadnia A., Nader N.D. (2025). Deep learning-based evaluation of panoramic radiographs for osteoporosis screening: A systematic review and meta-analysis. BMC Med. Imaging.

[17] Schwendicke F., Singh T., Lee J.H., Gaudin R., Chaurasia A., Wiegand T., Uribe S., Krois J., on behalf of the IADR e-Oral Health Network, the ITU WHO Focus Group AI for Health (2021). Artificial intelligence in dental research: Checklist for authors, reviewers, readers. J. Dent..

[18] Raza H., Ali M., Singh V.K., Wahjuningrum A., Sarig R., Chaurasia A. (2024). Segmentation of mental foramen in orthopantomographs: A deep learning approach. arXiv.

[19] Triantafyllopoulos G., Mitsea A., Rontogianni A., Korres D. (2023). Osteoporosis screening using dental panoramic radiographs and age at menarche. Diagnostics.

[20] Abdinian M., Milaei M., Soltani P. (2023). Digital panoramic radiography and CBCT as auxiliary tools for detection of low bone mineral density in post-menopausal women: A cross-sectional study. BMC Med. Imaging.

[21] Kavitha M.S., Asano A., Taguchi A., Kurita T., Sanada M. (2012). Diagnosis of osteoporosis from dental panoramic radiographs using the support vector machine method in a computer-aided system. BMC Med. Imaging.

[22] Sukegawa S., Fujimura A., Taguchi A., Yamamoto N., Kitamura A., Goto R., Nakano K., Takabatake K., Kawai H., Nagatsuka H. (2022). Identification of osteoporosis using ensemble deep learning model with panoramic radiographs and clinical covariates. Sci. Rep..

[23] Yen T.Y., Ho C.S., Chen Y.P., Pei Y.C. (2024). Diagnostic accuracy of deep learning for the prediction of osteoporosis using plain X-rays: A systematic review and meta-analysis. Diagnostics.

[24] Kiswanjaya B., Taufiq S.R., Syahraini S.I., Yoshihara A. (2025). The influence of age, sex, and mandibular morphometric parameters on cortical bone width and erosion: A panoramic radiography study. Front. Dent. Med..

[25] Minnema J., Wolff J., Koivisto J., Lucka F., Batenburg K.J., Forouzanfar T., van Eijnatten M. (2021). Comparison of convolutional neural network training strategies for cone-beam CT image segmentation. Comput. Methods Programs Biomed..

[26] Brown J.P., Josse R.G. (2002). 2002 clinical practice guidelines for the diagnosis and management of osteoporosis in Canada. CMAJ.

[27] Famili P., Cauley J., Suzuki J.B., Weyant R. (2005). Longitudinal study of periodontal disease and edentulism with rates of bone loss in older women. J. Periodontol..

