# Supplementary material for: AI-Based Automated Segmentation of the Mandibular Cortex and Mental Foramen for Radiomorphometric Measurement on Panoramic Radiographs: A Feasibility Study
Source: Diagnostics (Basel). 2026 Oct 19;16(20):3395. doi: 10.3390/diagnostics16203395 (PMC13703476; doi:10.3390/diagnostics16203395)

Supplementary Materials

Figure S1. Precision–recall curves of the YOLOv5s-seg model for the three classes (mandibular cortex, right mental foramen, left mental foramen) on the independent test set at an IoU threshold of 0.5. AP, average precision.

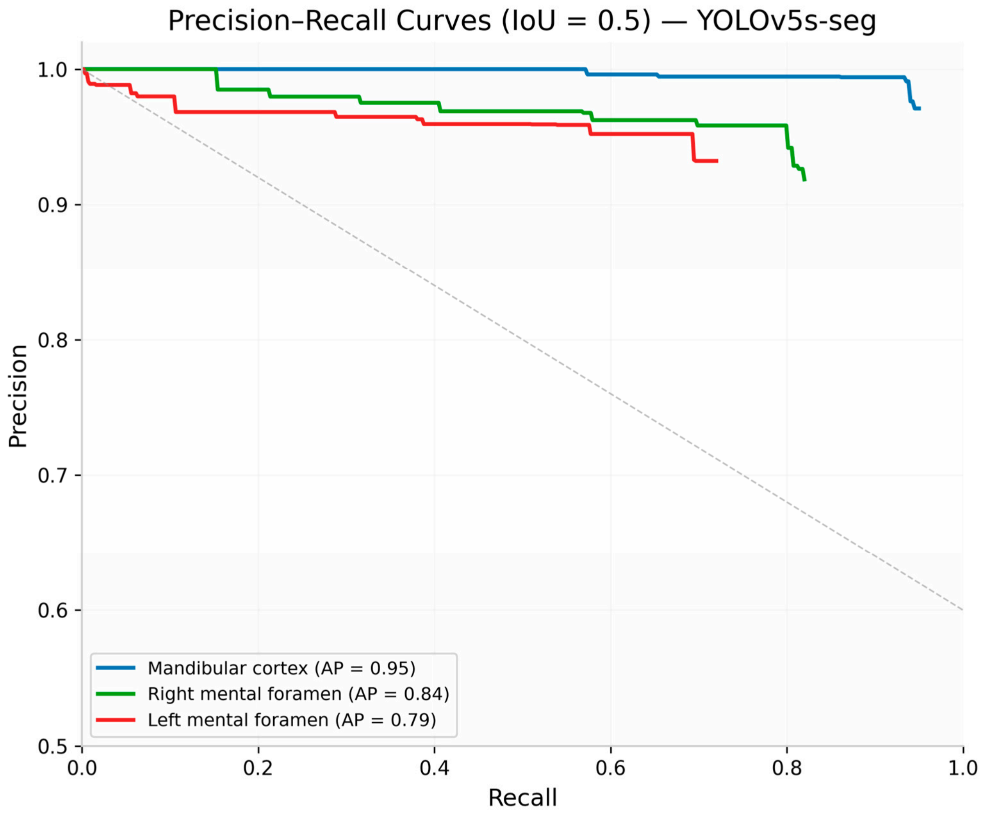

Figure S2. Training and validation learning curves over 500 epochs: box, segmentation, and classification losses for the training and validation subsets, together with object-level precision, recall, mAP@0.5, and mAP@0.5:0.95. The segmentation loss decreased steadily over the first ~300 epochs and plateaued thereafter, with no substantial divergence between training and validation loss.

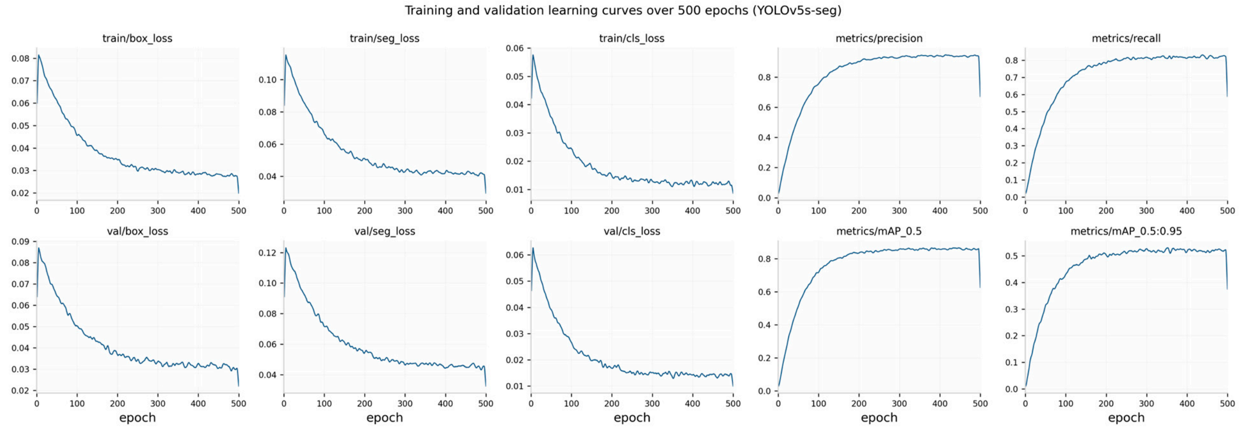

Supplement: Supplementary file 1 [file diagnostics-16-03395-s001.zip › diagnostics-4643967-supplementary.pdf]
